# Molecular epidemiology of HTLV-1 in Argentina

**DOI:** 10.1186/1742-4690-12-S1-P85

**Published:** 2015-08-28

**Authors:** María Pineda, María B Bouzas, Marcelo Golemba, Mirta Remesar, Paula Aulicino, Lilia Mammana, Luciana Tadey, Luisa Sen, A Mangano

**Affiliations:** 1 Lab. de Biología Celular y Retrovirus-CONICET, Hospital Garrahan, Buenos Aires, Argentina; 2 Unidad de Virología, Hospital Muñiz, Buenos Aires, Argentina; 3 Servicio de Hemoterapia, Hospital Garrahan, Buenos Aires, Argentina

## 

HTLV-1 is currently classified in 7 different subtypes (a-g) based on the nucleotide diversity of its LTR region. The Cosmopolitan subtype (a) has spread worldwide and is further divided into 5 sub-groups: Transcontinental (A), Japanese (B), West African (C), North African (D) and Peruvian (E). Different clusters has been described within the sub-group a including the Latin American cluster α and β and the South African cluster A. The aim of this study was to examine the molecular epidemiology of HTLV-1 in inhabitants of Argentina and explore its relationship with clinical features of the disease. HTLV-1 provirus DNA (LTR region of 672 bp) was amplified by nested PCR and sequenced from 23 isolates from residents of Argentina. Phylogenetic analysis showed that all sequences belonged to the Cosmopolitan subtype a, clustering in the Japanese B (n=1) and in the Transcontinental A (n=22). Among the latter, 1 clustered in the Latin American α (from Bolivia), 15 in the Latin American β from different geographic regions (9 Argentina, 2 Peru, 2 Paraguay, 1 Bolivia), 3 in the South African cluster A (1 Peru, 2 Buenos Aires), and 3 did not belong to any previously described clusters [2 Argentina, 1 Peru]. Among all the isolates, 4 belonged to symptomatic patients (3ATLL and 1 TSP). All ATLL cases belonged to the sub-type aA, clustering 2 within the Latin American β cluster and 1 with no specific cluster. The isolate from the TSP patient belonged to the South African cluster A. Her mother also belonged to this cluster but she was asymptomatic. In conclusion, most of the isolates belonged the Cosmopolitan subtype A and were from asymptomatic patients from the different regions. This is in accordance with previous reports that establish the major contribution of this sub-type to the circulation of the virus in South America. The only case of sub-type aB had no epidemiological link with Japanese descendants. Although, the phylogenetic classification of HTLV-1 has been associated with geographic localization of the infections, in this study we found a high frequency of isolates from Argentina and Peru (26%) not related to the Latin American cluster. This might reflect that the circulation of these viral types in South America is a recent phenomena.

